# Translation and Validation of the French Version of the Revised Green et al., Paranoid Thoughts Scale (R-GPTS) in Two Samples: Non-Clinical and Clinical Adults

**DOI:** 10.5334/pb.1134

**Published:** 2022-06-02

**Authors:** Alizée Latteur, Frank Larøi, Catherine Bortolon

**Affiliations:** 1 Psychology and Neuroscience of Cognition Research Unit, University of Liège, Belgium; 2 Department of Psychology, University of Oslo, Norway; 3 Norwegian Center of Excellence for Mental Disorders Research, University of Oslo, Norway; 4 Université Grenoble-Alpes, Laboratoire Interuniversitaire de Psychologie (LIP/PC2S), France; 5 C3R Centre Référent Réhabilitation Psychosociale et Remédiation, Centre Hospitalier Alpes-Isère, Grenoble, France

**Keywords:** Paranoia, psychometric properties, translation

## Abstract

Paranoia consists of unfounded beliefs that harm will be caused with intent to hurt the subject. Paranoid thoughts exist on a continuum of severity from severe forms in several psychological pathologies to milder forms in a significant minority of individuals of the general population (Freeman, 2007). It can be measured using several types of questionnaires. One recent questionnaire that measures paranoia in both clinical and non-clinical populations is the revised Green et al., Paranoid Thoughts Scale (R-GPTS) (Freeman et al., 2019). This questionnaire is an improved version of the Green et al., Paranoid Thoughts Scale (GPTS) (Green et al., 2008) and has excellent psychometric properties. In the present study, the R-GPTS was translated into French and the psychometric properties of the new French version were evaluated in a sample of the general population (N = 600) and in a clinical sample (N = 22). Confirmatory factor analysis supported the original two-factor structure (social reference and persecution subscales) of the R-GPTS. Evidence of excellent internal consistency of the R-GPTS was found. Furthermore, good convergent and discriminant validity was also found. Test-retest reliability showed significant positive correlations over a 1-month period. The findings discussed above were found in the non-clinical sample. Lastly, the R-GPTS revealed good preliminary criterion validity established from the comparison between the clinical and the non-clinical groups. In conclusion, the French version of the R-GPTS is a valid and reliable tool to measure paranoia in the general population. Due to the small sample size of the clinical sample, further studies are needed in order to confirm good psychometric properties in clinical populations, even though our preliminary findings are promising.

## Introduction

Paranoid beliefs are suggested to lie on a continuum with normality (Elahi et al., 2017; Freeman, 2007). Although social evaluative concerns and ideas of reference (e.g., people are talking about you) may be experienced by a significant minority of the general population, odder and less plausible paranoid ideas (e.g., delusions of persecution) are less often described in the general population when compared to clinical samples (Bebbington et al., 2013). Therefore, delusions can be seen as extreme manifestations of a normal and continuous phenomenon.

The Green et al. Paranoid Thoughts Scale (GPTS) was designed to measure the entire continuum of paranoia and includes a subscale for assessing ideas of social reference (16 items) and another subscale for persecutory ideas (16 items). It furthermore assesses three dimensions for each delusion: concern, conviction, and distress. The GPTS was designed to be used with both clinical and non-clinical participants, and moderate evidence of its measurement properties has been provided for both samples (Green et al., 2008; Ibáñez-Casas et al., 2015). According to a systematic review (Statham et al., 2019), the GPTS is the most valid and informative measure of paranoia so far. Nevertheless, the internal consistency and structural validity of the GPTS should be replicated in studies with larger samples. For instance, some items from the social reference subscale load onto the persecution subscale and other items from the persecutory subscale have highly correlated residuals (Freeman et al., 2019). Indeed, Freeman and colleagues (2019) ran a confirmatory factor analysis (CFA), and the original two-factor structure of the questionnaire could not be replicated.

Due to the questionable psychometric properties of the GPTS, Freeman et al. (2019) proposed a revised version of the questionnaire, that is, the revised Green et al., Paranoid Thoughts Scale (R-GPTS). The R-GPTS contains 18 items, eight for the ideas of social reference subscale and 10 items for the persecutory ideas subscale. As with the GPTS, the R-GPTS also measures different dimensions for each delusion, namely, degree of concern, conviction, and distress. The items from the social reference subscale that loaded onto the persecution subscale were removed, which helped provide a cleaner two-factor structure. Items from the persecution subscale with highly correlated residuals were also deleted. Another strength of the R-GPTS is that one can calculate a severity threshold. A score of 18 is used as a cut-off for persecutory delusions (Freeman et al., 2019). There is no cut-off score for the ideas of social reference subscale. However, from a score of 21, ideas of social reference may be considered as severe. The reduction in the number of items, compared to the GPTS (32 items to 18 items), is an additional advantage since it decreases the completion time of the questionnaire by almost half. Finally, the psychometric properties of the R-GPTS are excellent. The different items are very good at discriminating different levels of paranoia (that was measured with a discrimination parameter). Moreover, reliability is very good, with an alpha between 0.90 and 0.95 for the social reference subscale and an alpha between 0.90 and 0.97 for the persecution subscale. The reliability of the persecution subscale is particularly high at the severe end of the continuum of paranoia, indicating a valuable clinical tool. Freeman et al. (2019) concluded that this new questionnaire was better than the original GPTS.

The main goals of the present study were to translate the R-GPTS into French and to verify its psychometric properties. A confirmatory factor analysis was carried out in order to examine the adequacy of the original two-factor structure for the French version. The internal consistency, and construct validity (both convergent and discriminant validity), were also evaluated. Moreover, test-retest reliability and criterion validity were evaluated. Finally, in order to measure the criterion validity of the R-GPTS, a comparison was made between a sample from the general population and a clinical sample.

## Method

### Translation of the R-GPTS

We translated the R-GPTS by following Sousa and Rojjanasrirat’s (2011) guidelines. The original English version of the R-GPTS was translated into French by two independent bilingual individuals whose mother tongue was French. Both versions were compared in order to create a preliminary French version. Then, this French version was submitted to a back-translation procedure (i.e., the preliminary French version was translated back to English by a qualified translator). This English version was thereafter compared to the original version in order to verify the degree of similarity between both versions. After some changes, another French version was created and administered to 20 French-speaking persons who were asked to evaluate each item (on a Likert scale from 0 to 4) in terms of degree of clarity (they could also add any remarks or suggestions). Three French-speaking clinicians from local hospitals in Grenoble also completed this form. Based on this, some final adjustments were carried out, and a final French version of the R-GPTS was established by the authors (see Appendix).

### Sample size calculation

In order to calculate the necessary sample size of the non-clinical group, a website developed by Preacher & Coffman (2006) was used (http://www.quantpsy.org/rmsea/rmsea.htm). This website generates an R-code (to use with the computer software “R”) to determine the sample size required to obtain sufficient statistical power based on a covariance model using the average quadratic approximation error (RMSEA) that assesses the “Goodness of fit” model. Using this code with α = 0.05, df = 134, a power of 0.95, an alternative RMSEA of 0.08 (a RMSEA 0.08 is generally interpreted as low) and a zero RMSEA of 0.00 (based on Freeman et al. (2019)), the sample size indicated was 279. In order to compensate for potential loss (approximately 30%) and for subjects providing random answers (about 5%), 98 additional participants were added to this estimation, resulting in a total of 377 subjects. These calculations are based on models proposed by MacCallum et al. (1996).

Fewer participants were needed for the clinical group because they were not used in the confirmatory factor analysis. Therefore, as many clinical participants as possible were recruited. Moreover, we had no reason to believe the scale would behave differently in French clinical samples compared to British one recruited in the study by Freeman et al. (2019).

### Participants

Two samples were included in the present study: a non-clinical and a clinical sample.

Regarding the non-clinical sample, a total of 1083 French-speaking people took part in the online survey. Inclusion criterion included having French as their mother tongue (46 participants were excluded). Exclusion criteria included presenting reading difficulties (N = 53), having any psychological disorders (i.e., a depression, an anxiety disorder, a psychotic disorder, a personality disorder, or any other psychiatric disorder) (N = 168) and failing the validity checks (N = 18). Concerning this latter criterion, and based on previous studies (Kusztrits et al., 2020; Laloyaux et al., 2020), participants were excluded if they replied incorrectly to at least three validity items (see section “validity items”). Participants were also excluded if they did not answer all of the questions (N = 11) and if they answered in less than four minutes (N = 174) or in more than forty-five minutes (N = 13) for the entire questionnaires. Of the 1083 participants included in the survey, 483 were excluded after verification of the inclusion and exclusion criteria, resulting in 600 non-clinical participants being included in the study.

Concerning the clinical sample, 22 patients were recruited in local hospitals in the Grenoble area. They were included if they were experiencing persecutory delusions as determined by the psychiatrist or clinical psychologist who was treating the individual. Inclusion criteria included having French as their mother tongue. Exclusion criteria included presenting reading difficulties. Participants from both samples were not given any compensation for their participation in the study.

### Measures

#### Sociodemographic questionnaire

Participants first completed a sociodemographic questionnaire concerning their nationality, age, gender, educational level, familial situation, if they suffer from any psychiatric diseases, mother tongue and potential reading difficulties.

#### The Revised Green et al., Paranoid Thoughts Scale (R-GPTS)

The R-GPTS (Freeman et al., 2019) is a self-administered measure of paranoid ideation. The scale is composed of two subscales reflecting two dimensions of paranoid thinking, i.e., ideas of social reference and ideas of persecution. The R-GPTS contains 18 items: 8 items measuring ideas of social reference (e.g., “I spent time thinking about friends gossiping about me”) and 10 items measuring ideas of persecution (e.g., “I was certain people did things in order to annoy me”). Answers are rated on a response scale ranging from 0 (“Not at all”) to 4 (“Totally”). Participants are asked to complete the items in reference to the preceding month. The score for the ideas of social reference varies from 0 to 32, and the score for the ideas of persecution subscale varies from 0 to 40. The total score is calculated by adding up the scores of both subscales, with a high score indicating a higher tendency for paranoid thinking. The original English version of the R-GPTS possesses excellent psychometric properties, with an alpha between 0.90 and 0.95 for the social reference subscale and an alpha between 0.90 and 0.97 for the persecution subscale (Freeman et al., 2019).

#### The Peters et al. Delusional Inventory 21 items (PDI-21)

A French version (Verdoux et al., 1998) of the PDI-21 (Peters & Garety, 1996) was used to evaluate the convergent validity of the R-GPTS. The PDI-21 is a self-administered questionnaire that assesses delusional ideation. It contains 21 items. Answers are rated on a response scale ranging from 1 (“Never”) to 4 (“All the time”). Participants are asked to complete the items in reference to their usual state during the last five years. Scores can vary from 21 to 84, with a higher score indicating a higher tendency for delusion proneness. The PDI-21 has been found to measure delusion proneness adequately in the general population, with a Cronbach alpha coefficient of 0.88 (Peters et al., 1999).

#### The Cognitive-Perceptual subscale of the Schizotypal Personality Questionnaire-Brief (CP-SPQ-B)

A French version (Dumas et al., 2000) of the CP-SPQ-B (Raine, 1991) was administered in order to assess the convergent validity of the R-GPTS. The SPQ-B is a self-administered questionnaire that measures schizotypal traits and consists of 22 dichotomous (“Yes/No”) items. Participants are asked to complete the items in reference to their usual state. The CP-SPQ-B contains 8 items (e.g., “Have you ever had the sense that some person or force is around you, even though you cannot see anyone?”). The total score of the subscale ranges from 0 to 8, with a higher score indicating more cognitive-perceptual symptoms. A recent study with a large cross-cultural sample (N = 27000 from 12 countries) provided strong evidence in favour of a three-factor structure of the SPQ-B (Fonseca-Pedrero et al., 2018). The internal consistency of the SPQ-B is good for the three subscales and for the total score (with an α ranging from 0.79 to 0.9) (Cohen et al., 2010).

#### The Positivity Scale

The French version (in prep.) of the Positivity Scale (Caprara et al., 2012) was administered in order to measure the discriminant validity of the R-GPTS. The Positivity Scale is a self-administered questionnaire that measures positivity, that is, “a tendency to view and address life and experience with a positive outlook” (Caprara et al., 2012). The Positivity Scale is composed of eight items describing a positive view about the self (3 items), about others (1 item), about life (1 item) and about the future (3 items). Answers are rated on a Likert scale ranging from 1 (“Strongly disagree”) to 5 (“Strongly agree”). Participants are asked to complete the items in reference to their usual state. There are seven positively worded items (e.g., “I feel I have many things to be proud of”) and one negatively worded item (“At times, the future seems unclear to me”). The total score ranges from 8 to 40, with a higher score indicating a more positive way of thinking. The psychometric properties of the original version of the Positivity Scale are good, with an α of 0.75 (Caprara et al., 2012). In terms of the French version, the Cronbach’s alpha is 0.83 (in prep.).

#### Validity items

Six validity items were inserted throughout the different questionnaires in order to assess the validity of participants’ answers. These validity checks included: two attentional items in order to detect random completion or attentional lapses (e.g. “Please answer ‘agree’ to this question”), one item to detect potential lies (“I have always cheated in games”) from the Eysenck Personality Questionnaire-Revised (Eysenck et al., 1985) and three items to detect the simulation of psychotic experiences (e.g. “Have you ever experienced an hallucination that involved seeing white mice or other small animals?”) (Moritz et al., 2013). A total score out of six was calculated.

### Procedure

The non-clinical participants were recruited via social medias, posters and leaflets, and by word of mouth. The clinical sample was recruited in hospitals in the Grenoble area. For non-clinical participants, completion of all questionnaires took place online. A month later, they received a follow-up. The clinical sample only received the R-GPTS once. The study was carried out according to the code of ethics of the World Medical Association (Declaration of Helsinki) and was approved by the ethics committee of the Faculty of Psychology, Speech Therapy and Education Sciences (2021-007).

### Statistical analyses

Statistical analyses were conducted using the R-Studio software. Confirmatory factor analysis was carried based on previous studies (Krings et al., 2021) in order to examine the adequacy of the original two-factor structure for the French version. The following indices were selected in order to examine a model fit (Kline, 2005): Normed Chi-Square (χ^2^/df), with 2–5 indicating a good fit, Comparative Fit Index (CFI), Tucker-Lewis Index (TLI), Standardized Root Mean Square Residual (SRMR) and Bentler-Bonett or Normed Fit Index (NFI) with values above 0.95 suggesting a very good fit, and Root Mean Square Error of Approximation (RMSEA), with 0.01 indicating an excellent fit (0.05–0.08 indicating a reasonable error and acceptable fit).

In order to evaluate the internal consistency of the R-GPTS, the calculation of omega (ω) was applied. We used McDonald’s omega (ω) instead of Cronbach’s alpha (α) because it is known to be a more accurate measure of internal consistency (Dunn et al., 2014). It has less severe assumptions and fewer overestimation or underestimation risks (Kelley & Pornprasertmanit, 2016). A value equal to or greater than 0.70 shows satisfactory reliability (Béland et al., 2017).

For the next statistical analyses, we drew inspiration from Touzani & Salaani (2000). Since the data was not normally distributed, convergent and discriminant validities were examined using Spearman’s correlations. The R-GPTS is expected to measure a similar concept as the PDI-21 and the CP-SPQ-B (convergent validity), and thus a significant positive correlation is expected between the R-GPTS and PDI-21 and between the R-GPTS and CP-SPQ-B. The R-GPTS and the Positivity Scale should measure two distinct phenomena (discriminant validity) and they should therefore weakly correlate with each other.

Thereafter, Spearman’s correlations were employed in order to calculate the R-GPTS’s test-retest reliability over a 1-month period. Significant positive correlations between the first completion and the second completion are expected in order to indicate a good degree of test-retest reliability (Sousa & Rojjanasrirat, 2011).

Finally, independent sample t-tests were used in order to measure the criterion validity of the R-GPTS. Welch’s t-tests were used because variances of both samples were not equal. Significant differences in terms of R-GPTS scores were expected between the clinical and the non-clinical group. More specifically, it was predicted that the clinical group would score significantly higher on the R-GPTS (for both subscales and the total score).

Importantly, the analyses conducted for the confirmatory factor analysis, internal consistency, construct validity and test-retest reliability were carried out on data from the non-clinical sample. Data from the clinical sample was used to compute criterion validity.

## Results

### Sociodemographic characteristics of the sample

The mean age of the non-clinical respondents was 29.2 years (SD = 12.8, range 16–84), and they comprised 488 women (81.33%), 108 men (18%) and 4 with another gender (0.67%). Most of the participants had a master’s degree (43.17%), a bachelor’s degree (37.5%) or upper secondary level education (17.17%), while 3.5% had a PhD and 1.67% a lower secondary level education.

In the clinical group, the mean age was 30.6 years (SD = 7.91, range 17-46), and comprised 4 women (18.18%) and 18 men (81.82%). Most of the patients had a higher education (bachelor or master) (45.45%), or a professional high school (36.36%), while the others had a lower secondary level education (13.34%) or an upper secondary level education (4.55%).

### Confirmatory factor analysis

**Figure 1:**
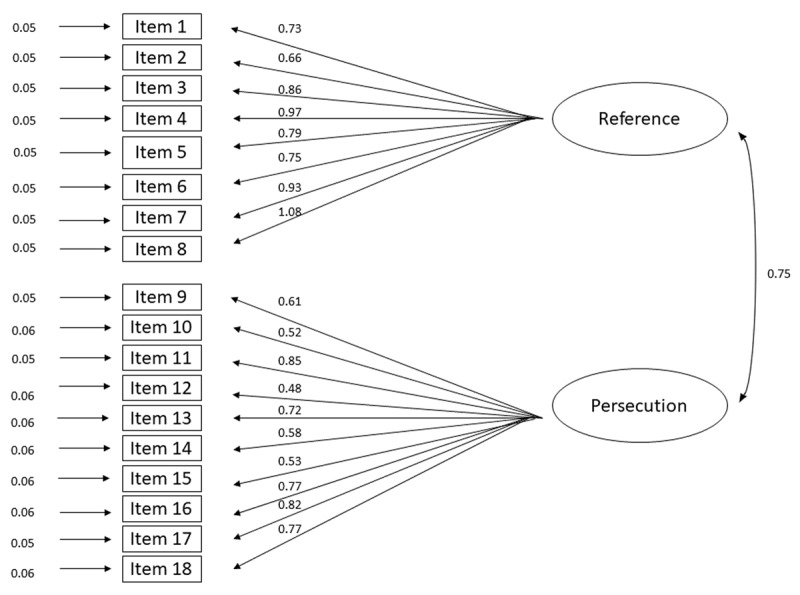
The two-factor model with Reference and Persecution as latent variables. *Note*: All manifest variables are depicted by rectangles. Measurement errors and factor loadings are indicated by single-head arrows. The correlation between the latent variables is illustrated with a double-headed arrow.

The two-factor model showed a good fit: CFI = 0.92, TLI = 0.91, SRMR = 0.049, χ^2^(134) = 359.598, *p* < 0.0001, χ^2^/df = 2.68. The RMSEA = 0.053 indicated a reasonable approximate fit. Conversely, the one-factor model showed a less adequate fit, CFI = 0.83, TLI = 0.80, SRMR = 0.07, χ^2^(135) = 623.633, *p* < 0.0001, χ^2^/df = 4.6, RMSEA = 0.078. Moreover, the AIC and BIC estimators indicated that the two-factor model (AIC = 27163.511; BIC = 27326.197) was superior to the one-factor model (AIC = 27662.368; BIC = 27820.658). Figure 1 illustrates the path diagram for the retained two-factor model.

### Internal consistency

For the total R-GPTS score, the omega was 0.94. The social reference subscale had an omega of 0.91 and the persecution subscale had an omega of 0.92. These scores are indicators of very high internal consistency.

### Convergent and discriminant validities

Table 1 presents Spearman’s correlations used to examine the convergent and discriminant validity of the French version of the R-GPTS. Our analyses revealed that all correlations were highly significant. As expected, the results showed positive correlations between the R-GPTS and the PDI-21 and between the R-GPTS and the CP-SPQ-B. The relationships for the total score were slightly higher when compared to the social reference and the persecution subscales. Finally, weak negative correlations were found between the R-GPTS and the Positivity Scale, with more negative correlations for the total R-GPTS score.

**Table 1:** Spearman’s correlations between total R-GPTS and R-GPTS subscales with the PDI-21, CP-SPQ-B and the Positivity Scale.


	SOCIAL REFERENCE SUBSCALE	PERSECUTION SUBSCALE	TOTAL R-GPTS

**PDI-21**	0.542*	0.577*	0.595*

**CP-SPQ-B**	0.397*	0.389*	0.429*

**Positivity Scale**	–0.291*	–0.258*	–0.304*


*Note*: * p < 0.001. PDI-21 = Peters et al. Delusional Inventory 21 items. CP-SPQ-B = Cognitive-Perceptual subscale of the Schizotypal Personality Questionnaire-Brief. R-GPTS = Revised Green et al., Paranoid Thoughts Scale.

### Test-retest reliability

All correlations between the first and the second completion were statistically significant (*p* < 0.001). Spearman’s correlations indicated high levels of test-retest reliability for the total score (*ρ* = 0.73) and moderate levels of test-retest reliability for the social reference subscale (*ρ* = 0.69) and the persecution subscale (*ρ* = 0.66).

### Criterion validity

Means and standard deviations are presented in Table 2. As expected, Welch’s t-test revealed significant differences between the clinical and the non-clinical groups on the total score, on the social reference subscale and on the persecution subscale. The clinical group showed higher results on all three scores of the R-GPTS. Both groups did not differ in age, however, the gender ratio was inverted. These results should be considered with caution due to the small size of the clinical group. A larger sample is required to be able to demonstrate the questionnaire psychometric properties for clinical samples. However, the present results are encouraging.

**Table 2:** R-GPTS in a non-clinical and in a clinical group.


	*GROUP*						

	NON-CLINICAL		CLINICAL				

**N**	600		22				

**Gender ratio (F/M)**	488/108		4/18				

	Mean	SD	Mean	SD	*t*	*p*	*d*

**Age**	29.2	12.8	30.6	7.91	0.81	0.426	0.13

**R-GPTS (total)**	12.42	13.07	24.2	21.39	2.56	0.009	0.664

**Reference Subscale**	7.74	7.22	11.8	9.56	1.96	0.031	0.477

**Persecution Subscale**	4.68	7.03	12.4	12.63	2.85	0.005	0.756


*Note*: R-GPTS = Revised Green et al., Paranoid Thoughts Scale. *d* = Cohen’s *d* score.

## Discussion

The R-GPTS seems to be the best questionnaire so far that measures paranoia in clinical and non-clinical populations. To the best of our knowledge, the questionnaire only exists in English (Freeman et al., 2019) and in Polish (Kowalski et al., 2020). A French translation of the R-GPTS would benefit both researchers investigating paranoia and clinicians wanting to assess it in their patients. In the present study, the R-GPTS was translated into French with a back-translation procedure and its psychometric properties were examined in both clinical and non-clinical groups.

Confirmatory factor analysis supported the original two-factor structure of the R-GPTS as presented in the original article (Freeman et al., 2019) with good fit indices (RMSEA, AIC and BIC). Moreover, results indicated that the internal consistency of the total score, the reference subscale, and the persecution subscale were very high and similar to the original English version (Freeman et al., 2019). The correlation coefficients of the convergent and discriminant validity of the total score, the reference subscale and the persecution subscale were also highly significant. As expected, positive correlations were found between the R-GPTS and the PDI-21 and between the R-GPTS and the CP-SPQ-B, suggesting good convergent validity. Furthermore, not only there was a weak, but also a negative, correlation between the R-GPTS and the Positivity Scale, indicating good discriminant validity.

In addition, the translated version of the R-GPTS revealed good test-retest reliability for the total score and moderate levels of test-retest reliability for the reference and persecution subscales. The findings described above were calculated based on the non-clinical sample. Finally, as expected, the comparison between the clinical and the non-clinical groups showed that the clinical group scored significantly higher on the total score and on both subscales of the R-GPTS compared to the non-clinical group, indicating good preliminary criterion validity. Altogether, the results revealed good to excellent psychometric properties of the French version of the R-GPTS in a non-clinical group. Further work is needed to confirm good psychometric properties of the R-GPTS in larger clinical samples, even though our preliminary findings are promising and corroborate the study by Freeman et al. (2019) who recruited a large sample of clinical and non-clinical participants.

Clinicians and researchers will therefore be able to use the French version of the R-GPTS in order to measure paranoid interpretations in general population samples. A strength of the present study is that multiple aspects of reliability and validity were examined in order to be as specific as possible in terms of the psychometric properties. Moreover, the sample size of the non-clinical sample exceeded the required sample size by 59 % (from 377 to 600), thus increasing the statistical power of our analyses. Thanks to the cut-offs identified by Freeman et al. (2019), French-speaking clinicians will be able to identify paranoia more precisely than before.

Due to the small sample size of our clinical group and to the unbalanced gender ratio, readers should be careful about the interpretation of our criterion validity section and thus our analyses should be replicated with a bigger sample size and with an equal gender ratio in order to confirm our conclusions in clinical samples. Moreover, the clinical and non-clinical samples are highly educated in comparison with the general population in Belgium (Census 2011). Despite this limitation, it should not be a problem as the French version of the R-GPTS corroborates the original study by Freeman et al. (2019).

## Conclusion

The French translation of the R-GPTS possesses good to excellent psychometric properties and can thus be considered as a valid and reliable tool to measure paranoia in the general population. Further studies are needed to confirm good psychometric properties of the R-GPTS in clinical populations.

## Appendix. The French Version of the Revised Green et al., Paranoid Thoughts Scale (R-GPTS)

Veuillez lire attentivement chacun des énoncés ci-dessous. Ceux-ci font référence à des pensées et à des sentiments que vous pourriez avoir expérimentés à propos d’autrui au cours du mois écoulé. Repensez au mois écoulé et indiquez l’intensité de ces sentiments sur une échelle de 0 (pas du tout) à 4 (tout à fait).

(Veuillez ne pas répondre aux énoncés en vous référant à des expériences que vous auriez éventuellement vécues sous l’influence de drogues).

Partie A:

**Table:**

	PAS DU TOUT		UN PEU		TOUT À FAIT

1. J’ai passé du temps à penser que des ami.e.s disent des ragots à mon sujet.	0	1	2	3	4

2. J’ai souvent entendu des gens parler de moi.	0	1	2	3	4

3. J’ai été contrarié.e par des ami.e.s et collègues qui m’ont jugé.e de manière critique.	0	1	2	3	4

4. Des gens ont clairement ri de moi dans mon dos.	0	1	2	3	4

5. J’ai beaucoup pensé au fait que des gens m’évitent.	0	1	2	3	4

6. Des gens ont fait allusion à mon égard.	0	1	2	3	4

7. J’ai cru que certaines personnes n’étaient pas ce qu’elles semblaient être.	0	1	2	3	4

8. Je me suis senti.e contrarié.e par les gens qui parlent de moi dans mon dos.	0	1	2	3	4

Partie B:

**Table:**

	PAS DU TOUT		UN PEU		TOUT À FAIT

1. Certaines personnes m’en veulent.	0	1	2	3	4

2. Des gens m’ont fixé.e du regard pour que je me sente menacé.e.	0	1	2	3	4

3. J’ai été certain.e que des gens ont fait des choses dans le but de m’énerver.	0	1	2	3	4

4. J’ai été convaincu.e qu’il y avait un complot contre moi.	0	1	2	3	4

5. J’ai été sûr.e que quelqu’un voulait me faire du mal.	0	1	2	3	4

6. Je ne pouvais pas m’empêcher de penser que des gens voulaient me rendre confus.e.	0	1	2	3	4

7. J’ai été en détresse car j’étais persécuté.e.	0	1	2	3	4

8. Je ne pouvais pas m’empêcher de penser aux personnes qui voulaient que je me sente mal.	0	1	2	3	4

9. Des gens ont fait exprès d’être hostiles envers moi.	0	1	2	3	4

10. J’ai été en colère parce que quelqu’un voulait me faire du mal.	0	1	2	3	4
